# Efficacy of removal of cariogenic bacteria and carious dentin by ablation using different modes of Er:YAG lasers

**DOI:** 10.1590/1414-431X20176872

**Published:** 2018-01-11

**Authors:** A. Baraba, L. Kqiku, D. Gabrić, Ž. Verzak, K. Hanscho, I. Miletić

**Affiliations:** 1 Department of Endodontics and Restorative Dentistry, School of Dental Medicine, University of Zagreb, Zagreb, Croatia; 2 University Clinic of Dental Medicine and Oral Health, Division of Prosthodontics, Restorative Dentistry, Periodontology and Implantology, Medical University Graz, Graz, Austria; 3 Department of Oral Surgery, School of Dental Medicine, University of Zagreb, University Hospital Center Zagreb, Zagreb, Croatia; 4 Department of Pediatric and Preventive Dentistry, School of Dental Medicine, University of Zagreb, University Hospital Center Zagreb, Zagreb, Croatia

**Keywords:** Dental caries, Er:YAG laser, Ablation, Real-time PCR, Thermography

## Abstract

The primary objective of this *in vitro* study was to evaluate the efficiency of removal of cariogenic bacteria and carious dentin by ablation using two lasers: fluorescence-feedback controlled (FFC) Er:YAG laser and different pulses of Er:YAG laser based on variable square pulse technology (VSPt). The secondary objective was to measure the temperature during laser ablation of carious tissue. Seventy-two extracted human molars were used in this study. Sixty teeth with carious dentin were randomly divided into four experimental groups according to the treatment for caries removal: group 1: 400 µs (FFC group); group 2: super short pulse (SSP group, 50 µs pulse); group 3: medium short pulse (MSP group, 100 µs pulse); group 4: short pulse (SP group, 300 µs pulse) and one positive control group with no treatment. Twelve teeth without carious lesion were used as a negative control group. After caries removal, swabs were taken with cotton pellets and real-time PCR analysis was performed. During caries ablation, a thermal infrared camera was used to measure the temperature changes. In all experimental groups, specimens were free of bacterial contamination after the treatment. In the SSP, MSP and SP groups, temperatures measured during caries ablation were significantly higher compared to temperatures in the FFC group (P<0.001). In this *in vitro* study, laser treatment for removal of carious dentin and cariogenic bacteria was an efficient treatment modality without causing excessive temperatures that might adversely affect pulp vitality.

## Introduction

The aim of caries excavation is to remove dentin contaminated by bacteria without removal of sound tooth structure, and maintain the vitality of the pulp (1). Caries lesions have two distinct substrates with different chemical composition and morphological structures: caries-infected and caries-affected dentin (2,3). The inner ‘infected dentin’ is a superficial and soft necrotic zone rich in bacteria, incapable of remineralization, with degenerated collagen fibrils that have lost their cross-links (4–6). If viable bacteria present in the infected dentin remain after caries removal, they may potentially release antigens into the pulp and cause a chronic inflammation reaction (7). Interestingly, well-sealed margins of the cavity seem to be more crucial for the long-term success of the restoration and pulp vitality than the presence of bacteria in the cavity since clinical follow-ups of bonded restorations placed over soft carious dentin showed that further progression of carious lesion can be arrested (8). However, caries-infected dentin showed extremely low cohesive strength as a result of low mineral content and changes in organic matrix (9), and consequently lower microtensile bond strength compared to sound and caries-affected dentin (10,11). Although thicker hybrid layers are obtained in caries-infected dentin, many dentin tubules remain completely free from tag formation (12–14). Due to this poor interaction during adhesive procedures, it is generally accepted that this layer needs to be removed prior to the placement of the restoration. Nevertheless, caries-affected dentin also produces lower bond strengths compared to normal dentin (15–18) due to lower mechanical properties and changes in chemical and morphological properties, and therefore this layer, which is free of bacteria or contains clinically insignificant number of bacteria, may be retained (4).

Different methods are described for caries removal, including rotary burs, hand instrument action, air abrasion, ultrasonic techniques, enzymes treatment, chemomechanical techniques and laser treatment (19). A laser commonly used for ablation of hard dental tissue is the Er:YAG laser. The erbium wavelengths coincide with the absorption peak of water and hydroxyapatite causing removal of hard dental tissue by microexplosions (20). Carious tissue contains even more water compared to healthy hard dental tissues and therefore the high absorption of the Er:YAG laser provides a selective and conservative caries removal without extending the preparation into the sound tooth structure. Introduction of the Er:YAG laser with variable square pulse technology (VSPt) enabled the use of very short, square-shaped pulses of adjustable duration. The pulse profile is controlled and ensures that the power within the pulse is approximately constant with all pulse energy being used up for ablation (21). However, according to the histological evaluation in a study by Medioni et al. (22), Er:YAG laser removed both infected and affected dentin. Another possibility to improve selective caries removal is the application of fluorescence feedback-controlled (FFC) Er:YAG laser, a combination of a diagnostic device and Er:YAG laser. With this system, the removal of dental hard tissues is controlled by the fluorescence signal from the tooth surface, induced by the red-infrared indium gallium arsenide diode laser (23). The excitation wavelength induces a fluorescence signal that has been assigned to protoporphyrin, a bacterial breakdown product (24). A sensor for the detection of fluorescence radiation of the dentinal tissue indicates whether any infected carious dentin is still present in the cavity, depending on the threshold level set when operating with FFC Er:YAG laser. The advantages of laser also include bactericidal properties from the high temperature (photothermal effect) during laser irradiation. The photothermal effect may be responsible for killing residual bacteria in cavities during caries ablation. However, the heat produced during laser ablation can cause thermal damage to irradiated substrates or the pulp in case of a temperature increase of more than 5.5°C, as shown in the *in vivo* study on monkeys by Zach and Cohen (25). Therefore, it is of outmost importance that these alternative techniques for caries removal are harmless for tooth pulp, in terms of thermal damage.

According to the literature available to the authors, there is no study comparing removal of cariogenic bacteria and carious dentin by measuring the temperature changes during caries ablation with different pulses of VSPt-based Er:YAG compared to FFC Er:YAG laser. Therefore, the primary objective of this *in vitro* study was to evaluate efficiency of removal of cariogenic bacteria and carious dentin by ablation with FFC Er:YAG laser and with different pulses of Er:YAG laser based on VSPt. The secondary objective was to measure the temperature during laser ablation of carious tissue.

## Material and Methods

### Study design

Seventy-two extracted human molar teeth were included in the study: 60 teeth with cavitated caries lesions extending into dentin, and 12 teeth without caries lesion. All molars were extracted for periodontal reasons at the Department of Oral Surgery, School of Dental Medicine, University of Zagreb. This experimental study was approved by the regional Ethical Review Board, University of Zagreb (106/09). Written informed consent was obtained from all participants to use their extracted teeth for scientific purposes. Inclusion criteria were teeth presenting cavitated caries lesions on the occlusal surface by visual examination and caries depth between the enamel-dentin junction and middle two-thirds of dentin examined on radiograph. Exclusion criteria were the presence of restorations or cavitations on other tooth surfaces. The tooth specimens were thoroughly cleaned of all residual debris using brushes and curettes and stored in saline for 2 weeks; saline was changed daily. All 60 teeth with carious lesions were randomly divided into four experimental groups (n=12) and one positive control group (n=12), while teeth without caries lesions were used as a negative control group (n=12). In the first experimental group, FFC Er:YAG laser (Key III, KaVo, Germany) was used. For the remaining three experimental groups, a second generation Fidelis Plus II Er:YAG laser (Fotona, Slovenia) with super short pulse (SSP, 50 µs), medium short pulse (MSP, 100 µs), and short pulse (SP, 300 µs), were used. For both lasers used all parameters were chosen according to the manufacturer’s instructions. During caries ablation, temperature changes were measured with an infrared thermal camera while real-time PCR analysis was used to determine the presence of cariogenic bacteria.

### Ablation procedures

All specimens were fixed with clamps during the caries removal treatment.

*Group 1* (*FFC group*). FFC Er:YAG laser was used with pulse energy of 350 mJ for the enamel cavity preparation and 250 mJ for the dentin treatment. The pulse duration was 400 µs with a repetition rate 10 Hz. The energy density for the enamel was 54.7 and 39.1 J·(cm^2^)^-1^·pulse for dentin^-1^. The contact handpiece (No. 2063, KaVo) with a spot size of 0.9 mm in diameter was used and the threshold level was set at 7 (10). The laser stopped emitting the laser beam when the fluorescence radiation of the dentinal tissue was beyond the previously set threshold level of 7 and this was considered the endpoint of the caries ablation. During laser treatment, hard dental tissues were kept wet with continuous water irrigation at the rate of 3 mL/min.

**Figure 1.**
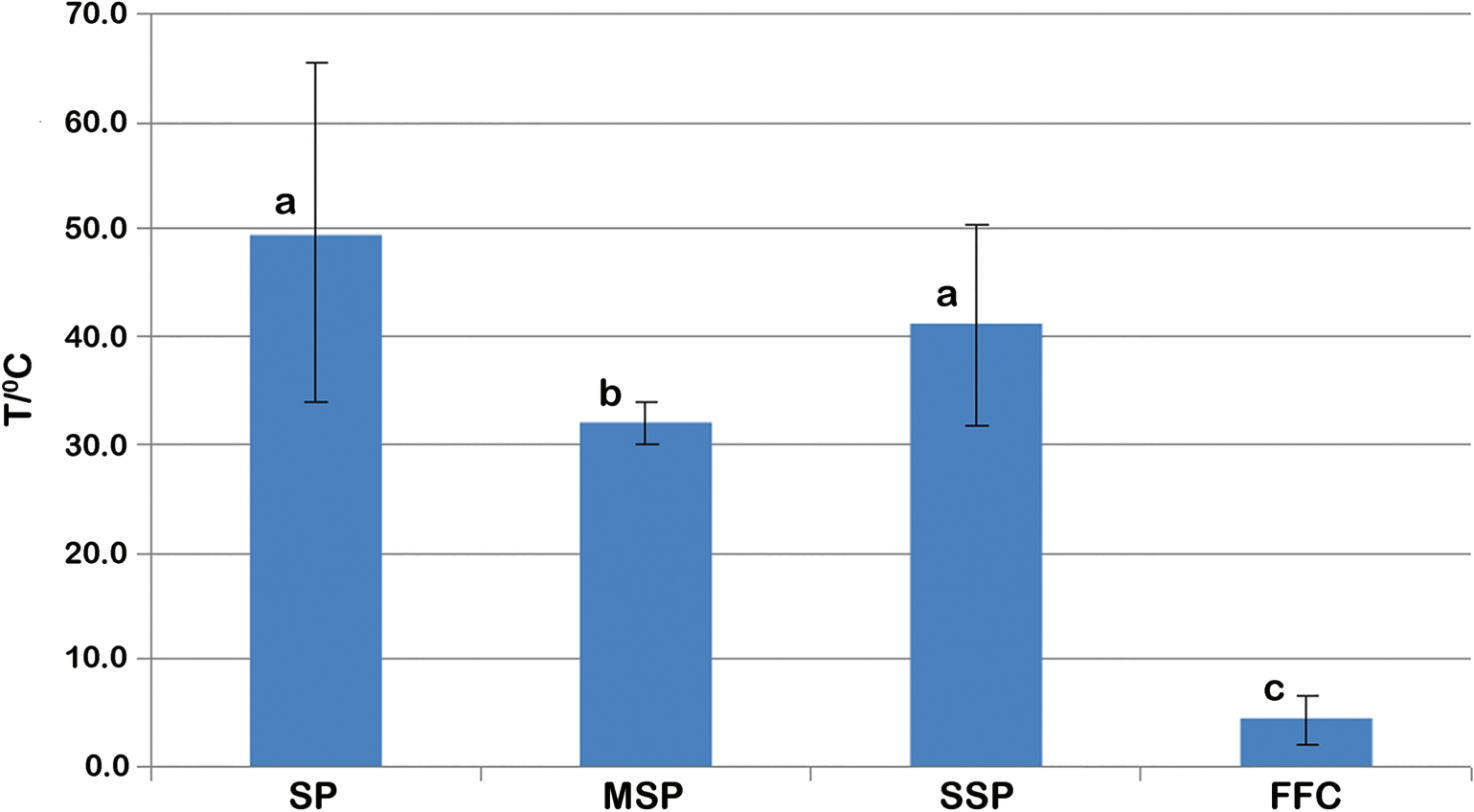
Average temperature (°C) and standard deviations for FFC, SP, MSP, and SSP groups. The temperatures were significantly higher in the SSP, MSP, and SP groups compared to temperatures in the FFC group (P<0.001). Temperature in the MSP group was significantly lower compared to SP (P<0.001), and SSP (P=0.002) groups. FFC: fluorescence feedback controlled; SP: short pulse; MSP: medium short pulse; SSP: super short pulse. Different letters indicate a statistically significant difference.

*Groups 2* (*SSP group*), *3* (*MSP group*) *and 4* (*SP group*). Cavities were prepared with a second generation Fidelis Plus II Er:YAG laser (Fotona) with super short pulse (SSP, 50 µs), medium short pulse (MSP, 100 µs), and short pulse (SP, 300 µs; Figure 1). The Er:YAG laser energy was delivered by a non-contact RO2-C handpiece with a spot size of 0.9 mm in diameter, under continuous water spray (3 mL/min) at a focus distance of 7 mm from the target. The pulse energy was 350 mJ for enamel and 250 mJ for dentin, with a pulse frequency of 10 Hz. The energy density for enamel was 54.7 and 39.1 J·(cm^2^)^-1^·pulse for dentin^-1^. The caries removal was performed under visual control by testing the hardness of the remaining tissue with a dental probe. The caries ablation procedure was considered complete after the dental probe induced a sharp sound and did not penetrate the dentin.

### Infrared thermography

During caries ablation at room temperature, temperature changes were measured with an infrared thermal camera. In the FFC group, a Thermosensorik GmbH camera was used (Thermosensorik GmbH, Geramny), and in SSP, MSP, and SP groups the ThermaCAM p45 (Flir Systems Inc., USA) was used for temperature measurement. Thermovision cameras were fixed and placed directly above the specimens at a distance of 20 cm.

### Real time PCR analysis

After caries ablation, samples were taken with a sterile cotton pellet from each experimental group and both positive and negative control groups. Cotton pellets were placed into Eppendorf tubes (Eppendorf, Germany), in 1 mL of phosphate buffer solution (PBS), and the contents of each tube were shaken to detach the samples of dentin from the cotton pellet. Real-time PCR analysis was used to investigate the presence of the gram-positive and gram-negative cariogenic bacteria in cavities after caries ablation (Table 1).

**Table 1.** Gram-positive and gram-negative cariogenic bacteria investigated in this study.

Gram-positive bacteria	Gram-negative bacteria
*Streptococcus mutans*	*Prevotella melaninogenica*
*Streptococcus sanguis*	*Fusobacterium nucleatum*
*Lactobacillus acidophilus*	*Porphyromonas endodontalis*
*Actinomyces israelli*	*Porphyromonas gingivalis*
*Peptostreptococcus micros*	

For the bacterial DNA isolation, as the first step in bacterial identification, the QIAamp DNA Stool Mini Kit (Qiagen GmbH, Germany) was used according to the manufacturer’s instruction. Real-time PCR analysis was performed by using Primerdesign™ Ltd. (Great Britain) genesis standards kits for *Lactobacillus acidophilus*, *Streptococcus sanguinis*, *Porphyromonas gingivalis, S. mutans*, *Actinomyces israelli, Prevotella melaninogenica*, *Fusobacterium nucleatum*, *Peptostreptococcus micros*, *P. endodontalis*, and *P. gingivalis*. The reaction mixture contained 20 µL: 10 µL of Precision™ Mastermixa (Primerdesign™ Ltd.), 1 µL of mixture of a primer and a probe (Primerdesign™ Ltd.), 4 µL of water and 5 µL of diluted DNA according to standard procedures. The PCR cycling conditions were performed as described in the amplification protocol of the PrimerDesign kit and cycling was performed in a LC480 (Roche Diagnostics, Austria). During PCR amplification, forward and reverse primers were hybridized to each bacterial DNA. A fluorogenic probe was included in the same reaction mixture, which consisted of a DNA probe labelled with a 5′-dye and a 3′-quencher. During PCR amplification, the probe was cleaved and the reporter dye and quencher were separated. The resulting increase in fluorescence was detected on a range of real-time PCR platform (LC480 (Roche Diagnostics). Positive control inside the specific kit was performed simultaneously during each PCR reaction, and RNAse/DNAse free water was used as negative control. The resulting data were averaged between the duplicates and then the proportions of each species were calculated against the universal assay primer/probe set (UniB) values of all species within each sample.

### Statistical analysis

Data was statistically analyzed with one-way ANOVA and Scheffe’s *post hoc* test with the level of significance set to 5%. The analysis was performed using Statistica 7.0 (StatSoft, USA).

## Results

Cariogenic bacteria were found in all specimens of the positive control group (Table 2) and not in specimens of the negative control group. In all laser experimental groups, specimens were free of the bacterial contamination after ablation procedure.

**Table 2.** Quantity of examined cariogenic bacteria DNA in negative control samples.

SM	SS	LA	AI	PM	FN	PeM	PE	PG
31.12	30.12	28.12	21.00	27.20	24.10	28.60	18.00	38.10
32.15	30.05	31.20	29.10	31.00	31.50	34.20	30.20	45.20
25.05	32.18	25.40	22.73	21.89	27.43	33.26	27.34	25.15
38.67	33.20	17.60	26.70	2.18	28.16	30.20	28.67	30.35
27.95	27.80	27.80	23.70	27.50	24.20	27.70	17.80	29.13
28.69	28.69	23.69	23.00	21.60	29.50	26.09	21.70	27.71
30.69	28.50	26.50	20.50	22.50	38.20	25.50	22.86	21.50
30.19	31.20	33.20	21.68	30.00	25.18	34.50	32.14	23.40
38.04	34.04	34.04	32.07	34.80	37.08	25.15	33.08	38.14
27.08	29.18	29.18	27.20	21.10	27.61	37.08	30.20	22.19
30.16	30.16	30.16	31.18	18.15	32.17	28.65	30.15	34.16
30.78	29.75	28.60	38.10	17.60	28.90	22.76	18.64	22.60

Data are reported as Ct values. Ct-cycle threshold: the lower the Ct value, the more DNA detected by a primer pair is present. SM: *Streptococcus mutans*; SS: *Streptococcus sanguis*; LA: *Lactobacillus acidophilus*; AI: *Actinomyces israelli*; PM: *Prevotella melaninogenica*; FN: *Fusobacterium nucleatum*; PeM: *Peptostreptococcus micros*; PE: *Porphyromonas endodontalis*; PG: *Porphyromonas gingivalis.*

Average temperatures for each group and the range of average temperatures for every specimen in experimental groups during caries ablation are shown in Figure 1. The temperatures were significantly higher in the SSP (41.2±9.3°C), MSP (32.0±1.9°C), and SP (49.5±15.7°C) groups compared to the temperatures in the FFC group (4.5±2.4°C; P<0.001). The temperature during caries ablation in the MSP group was significantly lower compared to SP (P<0.001) and SSP (P=0.003) groups. There was no significant difference in temperatures between SP and SSP groups (P=0.129).

## Discussion

The main purpose of this experimental study was to evaluate efficiency of removing cariogenic bacteria during ablation of carious tissue with two different lasers: FFC Er:YAG laser and VSPt based Er:YAG. In addition, the aim was also to measure the temperature changes during laser ablation of carious tissue. Qualitative and quantitative assessment of cariogenic bacteria of each sample was performed using RT PCR technique. Results showed that cavities in all experimental groups were free of bacterial contamination after laser-assisted caries ablation. Similar results were found only for FFC Er:YAG laser in studies that examined the amount of cariogenic bacteria after removal of carious tissue. These studies used different methods of evaluation of residual bacteria such as cultivation of dentin samples on culture medium for *S. mutans* and *L. species* (26) or histological staining for gram-positive and gram-negative bacteria (27). The mechanism of removing bacteria from the tooth cavity using laser technology is mechanical and photothermal. High temperatures cause changes in the cell wall and membrane of bacteria, along with denaturation of proteins and damage to nucleic acid, resulting in the death of bacteria (28). Furthermore, the photothermal effect is also induced after absorption of the laser beam by water, which causes microexplosions and breakup of the bacteria (29). Yamaguchi et al. (30) found that lipopolysaccharides in the cell membrane of gram-negative bacteria have a peak value of absorption of 2.92 µm, which is close to the wavelength of the Er:YAG laser. This specific laser removes 83.1% of lipopolysaccharides with a pulse energy of 100 mJ and frequency of 1 Hz. In this study, the pulse energy was 250 mJ, and the frequency 10 Hz, meaning that the bactericidal impact on the investigated gram-negative bacteria could be even more pronounced. It was also found that amines and amine groups that are present in bacteria also absorb the wavelength of the Er:YAG laser and this has a detrimental effect on bacteria due to the photochemical effect (31). Contrary to that, Valerio et al. (32) in their clinical study showed that affected dentin in the pulpal wall had similar amounts of *S. mutans* and *Lactobacillus sp*. comparing caries removal by Er:YAG laser and bur. In their study, microbiological analysis was performed by counting tested bacteria (32). However, inoculating cariogenic bacteria on culture medium in the laboratory might yield false positive results, because other bacteria, besides the tested ones, can grow on the same culture medium (33). The RT-PCR method, which was used in this study, is a more accurate method to detect clinically relevant cariogenic bacteria because it has a higher sensitivity and specificity in the detection of bacterial nucleic acids (34).

Temperature measurements showed the lowest values for the FFC Er:YAG group, probably due to a feedback control of the laser, which appeared to operate like a cut-off switch when infected dentin was eliminated (35) and stopped the laser from emitting energy intermittently. In this investigation, the threshold level was set at 7 according to the results of Krause et al. (26). They indicated that a fluorescence threshold level of 7 or 8 units can guide an Er:YAG laser to a complete removal of carious dentin (26). Interestingly, for Er:YAG based on VSPt the lowest value of average temperature was measured for the MSP group, while there was no significant difference between the SSP and SP groups. Diaci et al. (21) showed that shorter pulses with high energy enable a higher ablation rate of tissue than the diffusion of heat into the surrounding tissue. That causes less temperature rise compared to longer pulses like the SP used in this study. However, shorter pulses, like SSP, also decrease the time for cooling of hard dental tissue, which may explain the results of this study and the higher temperatures found for the shortest pulse. It should be emphasized that the infrared thermal camera measures the temperature in the center of the laser irradiation where the laser energy is maximal and produces large amount of heat, hence the temperature rise. These temperatures would be found in adjacent layers of dentin or in the pulp chamber.

Within the limitations of this study it was concluded that the Er:YAG lasers used in this study with different modes were efficient in removing cariogenic bacterial from infected dentin *in vitro*. In all treatment groups, cariogenic bacteria were completely removed. However, the lowest temperature rise was associated with the use of the FFC Er:YAG laser.

Based on this *in vitro* study, laser treatment for removal of carious dentin and cariogenic bacteria seems to be an efficient treatment modality without causing excessive temperatures, which might adversely affect pulp vitality.
